# Mitochondrial quality control modulating chondrocyte behavior and fate in knee osteoarthritis: mechanistic insights and therapeutic prospects

**DOI:** 10.3389/fimmu.2026.1671502

**Published:** 2026-02-25

**Authors:** Yi Li, Wen Zhong, Lan Li, Fengyuan Zhang, Xin Duan, Haibo Si

**Affiliations:** 1 Orthopedic Research Institute, Department of Orthopedics, West China Hospital, Sichuan University, Chengdu, China; 2 Department of Critical Care Medicine, West China Second Hospital, Sichuan University, Chengdu, China; 3 National Health Commission (NHC) Key Laboratory of Transplant Engineering and Immunology, Sichuan University, Chengdu, China; 4 Frontiers Science Center for Disease-related Molecular Network, West China Hospital, Chengdu, China; 5 Department of Orthopaedics, The Fifth People’s Hospital of Sichuan Province, Chengdu, China

**Keywords:** cartilage, cell behavior, cell fate, chondrocytes, mitochondrial quality control, osteoarthritis

## Abstract

Osteoarthritis (OA) is a highly prevalent and debilitating joint disorder that imposes a heavy burden on global public health due to its high incidence, prevalence, and disability rate, as well as the associated substantial healthcare costs. Early intervention is critical for OA management, yet current therapeutic options are limited by suboptimal efficacy, along with concerns regarding prosthetic lifespan and function in surgical treatment. While the complete etiology of OA remains elusive, cartilage degeneration is widely recognized as a core pathological feature of OA. A major barrier to optimizing OA therapeutic strategies is the lack of comprehensive insights into the underlying molecular mechanisms governing disease progression. Chondrocyte behavior and fate determination are pivotal to the onset and progression of OA: OA chondrocytes exhibit an imbalanced synthetic/catabolic profile, cluster formation, and autophagy dysregulation, accompanied by phenotypic alterations including hypertrophy and senescence. Additionally, multiple forms of chondrocyte death (apoptosis, chondroptosis, necrosis, necroptosis, autophagic cell death, pyroptosis, and ferroptosis) are implicated in driving OA development. Mitochondrial quality control (MQC), a cellular process encompassing redox homeostasis, mitophagy, mitochondrial dynamics (fusion and fission), and mitochondrial biogenesis, is essential for maintaining mitochondrial function and cellular homeostasis. Accumulating evidence indicates that MQC is closely involved in regulating chondrocyte behavior and fate in OA, and impaired MQC function may compromise chondrocyte viability and function, thereby promoting cartilage degeneration. Elucidating the MQC-mediated pathological mechanisms underlying abnormal chondrocyte behavior and fate in OA is expected to identify novel therapeutic targets for early-stage OA, thus providing new avenues for the development of more effective preventive and therapeutic strategies for this disorder.

## 1 Introduction

Osteoarthritis (OA) is a globally prevalent heterogeneous degenerative whole-joint disorder, with the knee as the most commonly affected site, and it has become a leading cause of physical disability worldwide, severely impairing patients’ quality of life and imposing a heavy burden on global healthcare systems (1). Global Burden of Disease 2021 data show the age-standardized prevalence of knee OA reached 4,294.27 per 100,000, with an 8.2% rise in disability-adjusted life years since 1990; the burden is higher in females, and aging, obesity and joint trauma are key risk factors, with early-onset OA increasing sharply (2). Current OA treatments are mostly palliative, failing to halt early progression, while late arthroplasty carries risks like prosthetic loosening, highlighting the urgency to elucidate OA’s multi-tissue pathogenesis and explore early targeted intervention targets (3, 4).

**Figure 1:**
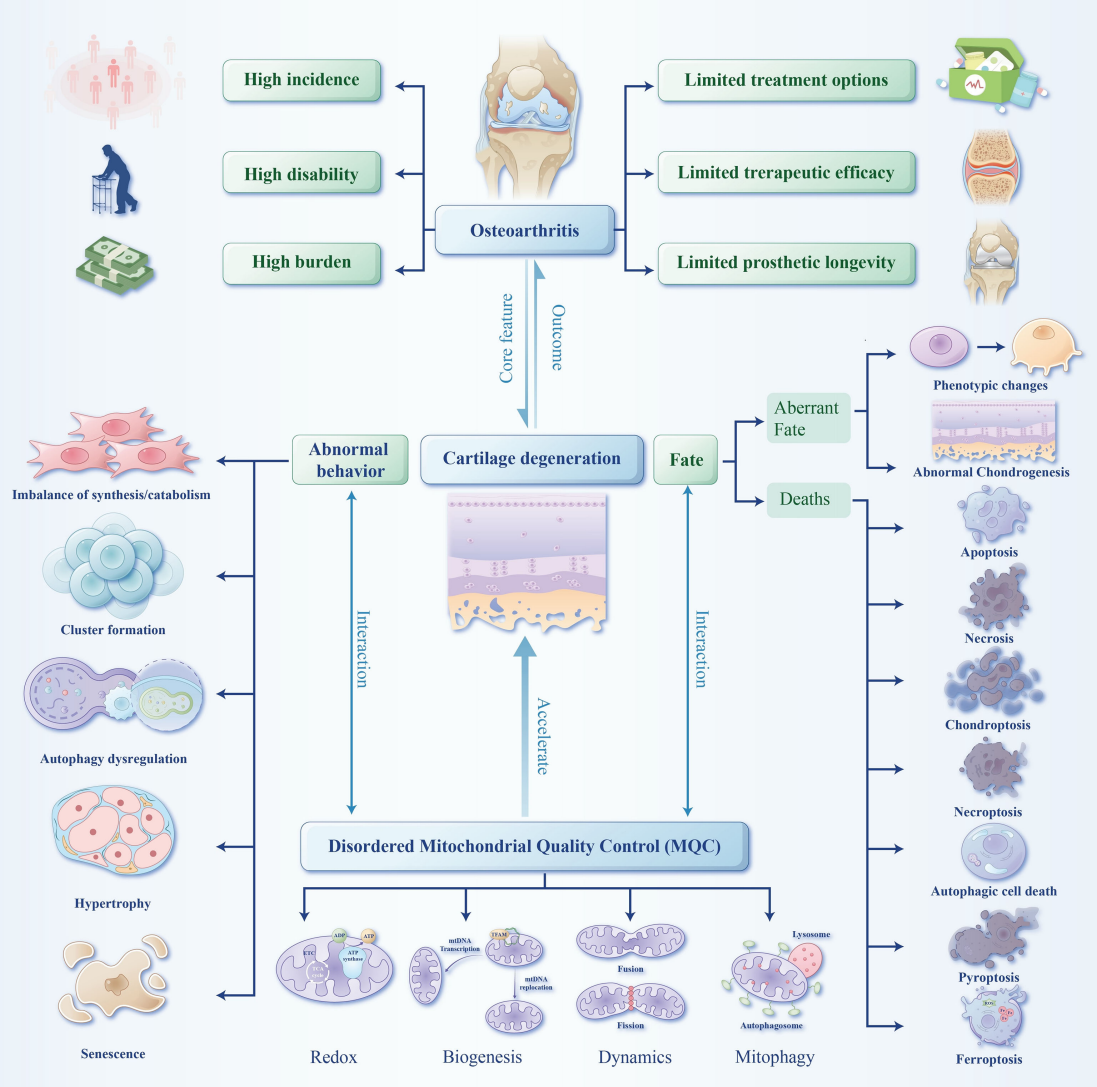
OA is highly prevalent, disabling and burdensome, with limited current treatments and prosthetic longevity. Centered on cartilage degeneration, disordered MQC accelerates this process by regulating chondrocyte aberrant behaviors and diverse cell death modalities.

OA is characterized by coordinated pathological changes of the whole joint rather than single tissue damage: the core is articular cartilage homeostasis disruption, with chondrocytes (cartilage’s only resident cells) losing function, reducing extracellular matrix synthesis and overactivating catabolic enzymes, leading to cartilage erosion (5, 6). This is accompanied by subchondral bone remodeling (early porosity, late sclerosis and trabecular disorder) and low-grade chronic synovitis, which form a vicious cycle accelerating cartilage degeneration (7). Chondrocyte dysfunction is the key link in OA progression; mitochondria supply energy for chondrocyte metabolism, and mitochondrial quality control (MQC) maintains mitochondrial function, with its dysfunction closely linked to OA chondrocyte pathological change (5, 8–10) Focusing on MQC, this review systematically summarizes the pathological mechanisms of MQC regulating the behavior and fate of OA chondrocytes, explores potential early intervention targets targeting MQC, and provides new ideas for precise OA treatment ***(*****Figure 1*****).***

## 2 Components and regulatory mechanisms of MQC

MQC is an integrated and dynamic regulatory network that plays an important role in maintaining mitochondrial integrity and functionality. It sustains ATP production through oxidative phosphorylation (18-fold more efficient than anaerobic glycolysis in chondrocytes) and regulates reactive oxygen species (ROS) generation, calcium homeostasis, and apoptosis, forming a fundamental basis for chondrocyte survival and functional maintenance (6, 11, 12). Herein, the components of MQC refer to the four core and interdependent functional modules that form the MQC regulatory network, including redox homeostasis, mitochondrial dynamics, mitophagy, and mitochondrial biogenesis. These four components are the essential structural and functional units of MQC, and they cooperate with each other to implement the full process of mitochondrial quality control—from ROS scavenging and mitochondrial morphological regulation to damaged mitochondrial clearance and *de novo* mitochondrial synthesis (11, 13). Among them, redox homeostasis acts as a central signaling hub to regulate crosstalk between various components, ensuring the coordinated operation of the entire MQC network. Impairment of this network is considered to be a contributing factor in the pathogenesis of OA, as it may induce chondrocyte phenotypic changes and cell death, which in turn can lead to cartilage matrix destruction (14, 15).

### 2.1 Redox homeostasis

As the core signaling node of MQC, its essence lies in the balance between the production and scavenging of ROS. ROS are mainly generated through electron leakage from complexes I and III of the electron transport chain (ETC), while their scavenging relies on antioxidant systems such as superoxide dismutase 2 (SOD2) and glutathione peroxidase (GPx) (16, 17). Under physiological conditions, ROS levels are strictly regulated; however, in OA chondrocytes, MQC defects lead to excessive ROS accumulation, triggering pro-catabolic responses and chondrocyte pathological changes (18). Notably, redox imbalance is closely associated with chondrocyte hypertrophy and senescence. For example, peroxiredoxin hyperoxidation is involved in OA progression, and ROS can also act as a ‘warning signal’ to initiate downstream MQC compensatory responses (19, 20).

### 2.2 Mitochondrial dynamics

It maintains mitochondrial network homeostasis through the dynamic balance between fission and fusion, which is tightly regulated by redox signals. Fission is mainly mediated by dynamin-related protein 1 (Drp1), while fusion is regulated by mitofusin 1/2 (Mfn1/2) and optic atrophy 1 (OPA1) (21). A mild increase in ROS can promote Drp1 phosphorylation, inducing selective fission of damaged mitochondria and facilitating subsequent mitophagy; on the contrary, Mfn1/2-mediated fusion can repair mild damage by integrating contents of healthy mitochondria (22). In OA, abnormal mitochondrial dynamics can induce chondrocyte senescence through chronic DNA damage and mitogen-activated protein kinase (MAPK) signaling activation, exacerbating chondrocyte dysfunction (23, 24).

### 2.3 Mitophagy

It is a selective autophagic process for clearing damaged mitochondria, mainly mediated by the PTEN-induced kinase 1 (PINK1)-Parkin pathway (24, 25). ROS-induced depolarization of mitochondrial membrane potential can stabilize PINK1 on the outer mitochondrial membrane, thereby recruiting Parkin to ubiquitinate mitochondrial proteins and mark damaged mitochondria for autophagic degradation. There is a synergistic regulatory relationship between mitophagy and mitochondrial fission—mitochondrial fragments generated by fission are more easily recognized by autophagosomes (25). In OA, mitophagy has a dual-edged effect; defects in mitophagy in cartilage tissue can exacerbate mitochondrial dysfunction and chondrocyte pathological changes, and are closely related to chondrocyte apoptosis and other forms of cell death (25).

### 2.4 Mitochondrial biogenesis

Driven by the peroxisome proliferator-activated receptor γ coactivator 1α (PGC-1α)/nuclear respiratory factor 1/2 (NRF1/2) pathway, it compensates for mitochondrial loss by synthesizing new functionally normal mitochondria (26, 27). ROS-activated AMP-activated protein kinase (AMPK) can phosphorylate PGC-1α, enhance its transcriptional activity, and upregulate the expression of mitochondrial DNA (mtDNA) replication and electron transport chain-related genes (27). This process forms a positive feedback loop with mitophagy—the metabolic intermediates released by mitophagy can further promote biogenesis (28). In OA, defects in mitochondrial biogenesis can promote pro-catabolic responses, while maintaining this process is crucial for chondrocyte homeostasis, making it a potential target for early OA intervention (29).

## 3 MQC dysregulation-mediated abnormal chondrocyte behaviors: the initial driver of OA

Articular cartilage, as the core load-bearing and buffering structure of synovial joints, its functional integrity is highly dependent on the dynamic homeostasis between chondrocytes and the extracellular matrix (ECM) (30–32). MQC, which comprises four core modules (redox regulation, mitochondrial dynamics, autophagy, and biogenesis), is presumably a potential molecular hub that contributes to the maintenance of the hypometabolic phenotype and functional homeostasis of chondrocytes (33). As the only functional cell type in articular cartilage, accounting for only 3% to 5% of the tissue volume, chondrocytes are responsible for the synthesis, turnover regulation, and damage repair of core ECM components (type II collagen, aggrecan) (28, 34). The hypoxic (oxygen partial pressure 1%-5%) and nutrient-poor microenvironment they reside in relies on MQC to maintain the stability of mitochondrial respiratory chain function, avoid excessive accumulation of ROS, and thereby ensure the balance of ECM synthesis and degradation (17, 35).

Recent studies have suggested that MQC dysfunction may serve as an initiating factor impairing this homeostasis; such dysfunction is likely to induce a range of abnormal chondrocyte behaviors via multi-dimensional regulation, potentially accelerating ECM degradation and the onset of cartilage degeneration, and thus may represent a core pathological link in the development of OA (36, 37).

### 3.1 MQC dysfunction may contribute to impaired ECM synthesis-degradation homeostasis

A key pathological consequence of MQC dysfunction may involve the impairment of ECM synthesis-degradation homeostasis in chondrocytes, a process that appears to be linked to the aberrant activation of ROS-mediated signaling pathways (38, 39). Under physiological conditions, MQC maintains the functional stability of electron transport chain complexes I/III through SIRT3-mediated deacetylation of mitochondrial proteins, inhibiting ROS leakage; meanwhile, it promptly removes damaged mitochondria through PINK1-Parkin pathway-mediated selective mitophagy, ensuring that catabolic enzymes such as matrix metalloproteinases (MMPs) and a disintegrin and metalloproteinase with thrombospondin motifs (ADAMTS) exist in a latent state, and their activities are precisely regulated by tissue inhibitors of metalloproteinases (TIMPs) (40).

In early OA, MQC dysfunction is first manifested as abnormal activation of mitochondrial dynamics imbalance, where Drp1-mediated mitochondrial fission is abnormally activated leading to mitochondrial fragmentation, accompanied by weakened Mfn1/2-mediated fusion function, directly damaging the integrity of the mitochondrial respiratory chain and triggering explosive accumulation of ROS (40, 41). Excessive ROS can not only directly oxidatively damage key ECM synthesis transcription factors such as Sox9, inhibiting the gene transcription of type II collagen (COL2A1) and aggrecan (ACAN); but significantly upregulate the expression and activation of MMP-2, MMP-13, and ADAMTS-5 by activating the NF-κB/p65 and p38-MAPK pathways (20, 42). Among them, MMP-13 can specifically degrade the triple helix structure of type II collagen, and ADAMTS-5 specifically hydrolyzes the chondroitin sulfate chains of aggrecan, jointly accelerating the destruction of the ECM skeleton (43).

Latest single-cell sequencing studies have shown that the downregulation of PGC-1α, a core MQC regulatory molecule, in early OA chondrocytes is highly correlated with the catabolic phenotype of chondrocytes (44, 45). PGC-1α deficiency can further weaken the compensatory capacity of chondrocytes by inhibiting mitochondrial biogenesis, exacerbating insufficient ECM synthesis; meanwhile, complement component C5a in synovial fluid can synergize with MQC dysfunction to amplify the activation effect of the NF-κB pathway by activating the C5aR1 receptor on the surface of chondrocytes, forming a vicious cycle of ‘MQC dysfunction-ROS accumulation-complement activation’ and further enhancing the catabolic response (43, 46).

### 3.2 MQC dysfunction mediates abnormal chondrocyte clustering

Abnormal chondrocyte clustering is another important pathological behavior mediated by MQC dysfunction, whose formation is closely related to mitochondrial metabolic reprogramming (47). In OA cartilage, MQC dysfunction leads to abnormal mitochondrial tricarboxylic acid (TCA) cycle function, accumulation of metabolic intermediates such as α-ketoglutarate and succinate, which affect epigenetic modifications by regulating the activity of histone demethylases, driving abnormal proliferation and aggregation of chondrocytes. Specifically, ROS accumulation caused by MQC defects can activate the ERK1/2 and PI3K-Akt pathways; the former promotes the expression of cyclin D1 to drive cell proliferation, while the latter enhances cell migration ability, jointly promoting the formation of clustered structures (47, 48).

Single-cell sequencing studies have suggested that although clustered chondrocytes highly express autophagy-related proteins ULK1, Beclin-1, and LC3II, mitophagic flux is blocked, making it impossible to effectively clear ROS-damaged mitochondria, leading to continuous ROS accumulation and forming a vicious cycle of ‘mitochondrial damage-ROS outburst-abnormal proliferation-autophagic disorder.’ (46, 47) More importantly, such clustered cells have undergone phenotypic malignant transformation, highly expressing hypertrophy markers such as Runx2, alkaline phosphatase (ALP), and type X collagen (COL10A1), while excessively secreting FGF2 and MMP-13. The ECM synthesized by them is mainly composed of fibrocartilage components, lacking the elasticity and load-bearing function of normal articular cartilage, and instead accelerating the fibrotic degeneration of cartilage tissue (49).

In addition, recent studies have found that clustered chondrocytes can recruit synovial macrophage infiltration by paracrine inflammatory factors such as IL-6 and MCP-1, further amplifying the local inflammatory microenvironment of the joint and enhancing the damaging effect of MQC dysfunction on chondrocytes (50).

### 3.3 Mitophagic disorder: the core node connecting MQC dysfunction and chondrocyte abnormalities

Mitophagic dysfunction may represent a key node linking MQC impairment to a range of chondrocyte abnormalities, and its functional status seems to be closely associated with the initiation and progression rate of OA (22). In the early stages of OA, chondrocytes are thought to initiate a compensatory response by activating mitophagy, clearing a subset of damaged mitochondria, and preserving the balance of ECM synthesis; animal studies have indicated that targeted knockout of autophagy-related genes Atg5 or Beclin-1 in chondrocytes can result in the accumulation of mitochondrial damage, ROS overproduction, and a marked acceleration in the progression of age-related OA (51).

However, with the continuous deterioration of MQC function, inflammatory factors such as IL-1β and TNF-α inhibit autophagy initiation by activating the mTORC1 pathway, and ROS can directly oxidatively damage autophagosomal membrane proteins, hindering the fusion of autophagosomes and lysosomes, resulting in complete blockage of autophagic flux (52). The massive accumulation of damaged mitochondria not only exacerbates ROS production, but also activates the caspase-9-dependent intrinsic apoptotic pathway by releasing cytochrome C, and promotes NLRP3 inflammasome activation to trigger pyroptosis, further reducing the number of functional chondrocytes (53).

In summary, MQC dysfunction may initiate and promote the process of cartilage degeneration by mediating a cascade reaction of ‘ECM synthesis/degradation imbalance-abnormal clustering-autophagic disorder-cell death,’ among which ROS-mediated signaling pathway activation and mitochondrial metabolic abnormalities are important regulatory links. Targeted repair of MQC function, such as activating the PGC-1α pathway to enhance mitochondrial biogenesis and regulating Drp1 activity to restore mitochondrial dynamics balance, may help alleviate abnormal chondrocyte behaviors, thereby offering potential precision molecular targets for the development of early OA interventions.

## 4 MQC dysregulation-induced chondrocyte phenotypic remodeling: a key link in OA progression

Under physiological conditions, articular chondrocytes maintain a stable hypometabolic phenotype to sustain cartilage homeostasis, a state that is tightly orchestrated by the coordinated actions of MQC machinery (54). Emerging evidence suggests that the onset and progression of OA are accompanied by MQC dysfunction, which may disrupt mitochondrial homeostasis and trigger aberrant phenotypic remodeling of chondrocytes—most notably the induction of hypertrophy and senescence (55). These two pathological phenotypes are thought to interact synergistically, potentially ECM degradation and cartilage destruction, and thus may represent a key mechanistic link contributing to OA pathogenesis. Accumulating preclinical and clinical evidence has begun to delineate the regulatory role of MQC dysfunction in chondrocyte phenotypic remodeling, providing tentative insights for the development of targeted OA interventions (49).

### 4.1 MQC dysfunction may contribute to chondrocyte hypertrophy

Chondrocyte hypertrophy, a typical phenotypic aberration in OA, is characterized by the upregulation of hypertrophic markers such as Runx2 and type COL10A1-a cellular state that has been closely correlated with MQC dysfunction in both *in vitro* and *in vivo* models (55, 56). Growing experimental data implicates mitochondrial dynamics imbalance is an initiating factor for chondrocyte hypertrophy: in early OA, abnormal activation of Drp1-mediated mitochondrial fission leads to mitochondrial fragmentation, and the impaired Mfn1/2-mediated fusion function fails to repair damaged mitochondria, resulting in ROS overaccumulation. Excessive ROS activates the p38-MAPK pathway in chondrocyte models, his pathway can phosphorylate Runx2 to enhance its transcriptional activity, thereby promoting the expression of MMP-13 and COL10A1, processes that are thought to accelerate ECM degradation and cartilage calcification.

In genetic models, PGC-1α deficiency has been shown to inhibit mitochondrial biogenesis, further exacerbating mitochondrial dysfunction and ROS accumulation; this cascade appears to form a feed-forward loop with chondrocyte hypertrophy, though direct causal evidence in human OA remains to be fully established (57, 58). Additionally, mitophagic deficiency, a key facet of MQC dysfunction, can lead to the accumulation of damaged mitochondria; preclinical data suggests that this accumulation activates the hypoxia-inducible factor 2-alpha (HIF-2α) pathway to induce chondrocyte hypertrophic differentiation, which may contribute to OA progression (59).

### 4.2 MQC dysfunction as a putative driver of chondrocyte senescence

Chondrocyte senescence is another critical phenotypic change in OA progression, and MQC dysfunction has emerged as a key candidate driver of this process. Under conditions of aging or mechanical stress—both well-established OA risk factors—MQC dysfunction may trigger mitochondrial ROS overproduction and mtDNA damage. Accumulated mtDNA fragments have been shown to activate the cyclic GMP-AMP synthase-stimulator of interferon genes (cGAS-STING) pathway in chondrocyte models, promoting the expression of senescence-related markers such as p16INK4a and p21Cip1/Waf1, and inducing a senescent phenotype (60).

Senescent chondrocytes typically exhibit reduced anabolic activity, enhanced catabolic capacity, and secretion of a diverse array of senescence-associated secretory phenotype (SASP) factors (58, 61). Notably, MQC dysfunction can further amplify the SASP response: impaired mitochondrial autophagy leads to continuous activation of the NF-κB pathway by damaged mitochondria, promoting the secretion of IL-6, TNF-α and other SASP factors, which not only maintain the senescent state of chondrocytes in an autocrine manner, but also induce senescence of surrounding normal chondrocytes in a paracrine manner, expanding the pathological damage (62).

### 4.3 Synergistic interaction between hypertrophy and senescence mediated by MQC dysfunction

MQC dysfunction is widely regarded as a key upstream regulator that may promote the coordinated development of chondrocyte hypertrophy and senescence in the early stages of OA. A central molecular link underlying this crosstalk is thought to be the activation of p38 MAPK by mitochondrial ROS (mtROS) (63). Recent studies have confirmed that mtROS resulting from MQC failure specifically triggers the phosphorylation of p38 MAPK at its activation loop residues Thr180 and Tyr182–a critical step for its full activation (64, 65).

Once phosphorylated and activated, p38 MAPK translocates to the nucleus, where it enhances the transcriptional activity of Runx2 by phosphorylating it at key sites such as Ser301 and Ser319. This leads to the upregulation of hypertrophy markers, including collagen type X and MMP-13 (66). Concurrently, activated p38 promotes the transcription of SASP factors such as IL-6 and p16INK4a, thereby accelerating cellular senescence (24). These two processes form a vicious cycle: SASP-derived matrix-degrading enzymes cleave type II collagen, and the resulting fragments bind to DDR2 to further promote hypertrophy, while hypertrophic chondrocytes themselves generate more mtROS, amplifying p38 MAPK activation and senescence (42). Therefore, targeting the mtROS-p38 MAPK (Thr180/Tyr182) axis represents a promising strategy.

While these preclinical findings highlight the mtROS-p38 MAPK (Thr180/Tyr182) axis as a promising target to disrupt this detrimental feedback loop, it is important to note that translational challenges remain. For example, the specificity of p38 MAPK inhibitors in chondrocytes, potential off-target effects on synovial or subchondral bone cells, and the lack of long-term safety data in human OA cohorts represent critical barriers to clinical application. Moreover, most mechanistic studies to date rely on animal models or immortalized chondrocyte lines, and direct causal evidence linking MQC dysfunction to concurrent hypertrophy and senescence in human OA cartilage is still emerging.

## 5 MQC collapse-induced chondrocyte death: a pivotal event in OA progression

With the progression of OA to advanced stages, the compensatory capacity of MQC is thought to become substantially depleted, which may contribute to the breakdown of mitochondrial homeostasis. As a key upstream factor of OA deterioration, MQC collapse triggers massive chondrocyte death through multiple pathways, including apoptosis, pyroptosis, ferroptosis and necroptosis (64, 67–69). (**Table 1**) Since chondrocytes have limited regenerative capacity, their massive loss directly impairs the structural integrity and functional stability of articular cartilage, forming a vicious cycle with ECM degradation and joint inflammation, potentially accelerating OA pathogenesis (70).

**Table 1:** The general features of chondrocyte death in osteoarthritis.

Type of death Morphological features	Apoptosis	Chondroptosis	Necrosis	Nercoptosis	Autophagic death	Pyroptosis	Ferroptosis
Cell morphology	Shrinkage	Shrinkage	Swelling (oncosis)	Shrinkage	Shrinkage	Swelling	Shrinkage
Cytoplasmic membrane	Blebbing but intact	Vesicle blebs	Disrupted	Disrupted	Participate to autophagosome formation	Disrupted	Disrupted
Cytoplasm	Densed	Densed	Granulation	Decomposition	Decomposition	Decomposition	Decomposition
Increase of organelles	No	prominent expansion of the rough endoplasmic reticulum (RER) and the Golgi apparatus	No	No	No	No	No
Nucleus	Fragmentation into apoptotic bodies	Convoluted nucleus Nuclear condensation	Nuclear condensation (pyknosis)	Decomposition	Intact until late stages	Nuclear condensation (pyknosis)	Intact until late stages
Chromatin	Marginal condensation at the nucleus membrane and aggregate in larger masses	Patchy condensation throughout the nucleus	Fragmented	Fragmented	Absence of condensation	Fragmented	Fragmented
DNA	Intranucleosomal cleavage-DNA laddering	Cleaved	Random cleavage DNA Smear	Random cleavage DNA Smear	DNA fragmentation occurs very late	Random cleavage DNA Smear	Random cleavage DNA Smear
Apoptotic bodies	Yes	Cellular remnants and vesicules	Cell explosion	Cell explosion	No	No	No
Autophagic vacuoles	No	Frequent	No	No	Abundant	No	No
Rough endoplasmic reticulum		Prominent expansion	Swelling	Swelling	Protuding、engulfing	Swelling	Swelling
Golgi apparatus	No increase	Increase in early stages	No increase	No increase	Enlargement	No increase	Enlargement
Behavioral characteristics							
Inflammation	No	Not precised	Yes	Yes	No	Yes	Yes
ER	No enlargement	Increase in amount and expansion of lumen	No enlargement	No enlargement	Enlargement	No enlargement	Enlargement
Release of cellular contents into intracellular space	No	No	Yes	Yes	No	Yes	Yes
Elimination	Phagocytosis of apoptotic bodies	Phagocytosis-independent self destruction	General lysis	General lysis	Auto-elimination	General lysis	General lysis
Occurs in physiological situations	Yes	Yes	Yes	Yes	Yes	No	Yes
Mechanistic features							
Lysosomal enzyme	Inside apoptotic bodies	Inside cytoplasmic ‘islands’ or autophagic vacuoles	Leakage	Leakage	Inside autophagic vacuoles	Leakage	Leakage
Caspase-dependent	Yes	Yes	No	No	No	No	No
Energy-dependent	Yes	Not precised	No	No	Yes	No	No

A growing body of evidence suggests that MQC collapse-mediated chondrocyte death is a pivotal pathological event in OA deterioration. Targeting MQC to block abnormal chondrocyte death has emerged as a promising therapeutic strategy for advanced OA (71). This section focuses on the regulatory mechanisms of MQC collapse in various forms of chondrocyte death and their synergistic role in OA exacerbation.

### 5.1 Apoptosis and chondroptosis

Apoptosis is a well-characterized programmed cell death process and a major form of chondrocyte loss in OA. MQC collapse tightly regulates its occurrence by disrupting mitochondrial dynamics and activating the intrinsic apoptotic pathway (72). Recent studies have shown that MQC collapse-induced hyperactivation of Drp1 and inhibition of Mfn2 lead to mitochondrial fragmentation and loss of membrane potential, promoting the opening of mitochondrial permeability transition pore (mPTP) and the release of cytochrome c. The formed apoptosome activates the caspase cascade, ultimately inducing apoptosis (73).

Moreover, mitochondrial ROS burst amplifies apoptotic signals through phosphorylating JNK/p38-MAPK pathways, upregulating Bax and downregulating Bcl-2. Chondroptosis, a chondrocyte-specific apoptotic variant, is closely associated with MQC collapse. 2025 research revealed that MQC collapse disrupts mitochondrial-associated ER membranes (MAMs), causing Ca²^+^ imbalance and ER stress, which upregulates caspase-1 activity. Activated caspase-1 promotes IL-1β secretion and induces chondroptosis characterized by mitochondrial swelling, which amplifies joint inflammation and forms a positive feedback loop with MQC collapse (72, 74).

### 5.2 Necrosis and necroptosis

Necrosis, a passive cell death caused by severe damage, and necroptosis, a regulated necrotic process, are both involved in MQC collapse-mediated chondrocyte loss. Severe MQC collapse triggers catastrophic mitochondrial dysfunction, leading to a sharp decrease in ATP production and excessive ROS accumulation, which causes necrosis by damaging cell membranes and releasing damage-associated molecular patterns (DAMPs) to induce synovial inflammation (75). Necroptosis, regulated by the RIPK1/RIPK3/MLKL pathway, is a significant programmed necrotic form in OA.

A 2024 study suggests that energy metabolism disorders and ROS accumulation associated with MQC breakdown may activate this pathway by promoting RIPK1/RIPK3 phosphorylation and necrosome formation, potentially leading to MLKL-mediated membrane pore formation and subsequent cell rupture (76, 77). However, the precise role and regulatory mechanisms of necroptosis in OA pathogenesis remain to be elucidated.

### 5.3 Autophagic cell death

Autophagic cell death (ACD), a programmed cell death dependent on autophagy machinery, is increasingly recognized as an important form of chondrocyte death in OA. MQC and autophagy have bidirectional crosstalk: mitophagy, a core component of MQC, is a specific autophagy for clearing damaged mitochondria. Under physiological conditions, moderate autophagy maintains chondrocyte homeostasis, but persistent MQC collapse in advanced OA leads to excessive autophagy and transforms it into pathogenic ACD (78).

2024 research demonstrated that MQC collapse activates the AMPK/mTOR pathway by accumulating damaged mitochondria, inhibiting mTOR to trigger excessive autophagy (78). Meanwhile, mitochondrial ROS upregulates autophagy-related genes (Beclin-1, LC3-II) via FOXO3a (59). Moreover, MQC collapse impairs autophagosome-lysosome fusion, causing immature autophagosome accumulation and aggravating ACD. Notably, impaired mitophagy promotes excessive autophagy, and excessive autophagy further inhibits mitophagy by degrading PINK1/Parkin, forming a vicious cycle.

### 5.4 Pyroptosis

Pyroptosis, an inflammatory programmed cell death dependent on caspases and gasdermin proteins, plays a key role in OA inflammation amplification. MQC breakdown appears to act as a critical upstream factor implicated in the induction of pyroptosis. Recent studies indicated that MQC collapse-induced accumulation of damaged mitochondria releases mtDNA, which acts as an endogenous danger signal to activate the NLRP3 inflammasome (79). The assembled inflammasome activates caspase-1, which cleaves gasdermin D (GSDMD) to form membrane pores and promotes IL-1β/IL-18 maturation. This leads to cell swelling rupture and inflammation amplification (80). The expression levels of NLRP3, caspase-1, and GSDMD-N in OA cartilage are positively correlated with OA severity. Preclinical evidence indicates that restoring MQC function via the activation of mitophagy may reduce mtDNA release and inhibit pyroptosis, a strategy that has been proposed as a potential therapeutic target for OA.

### 5.5 Ferroptosis

Ferroptosis, an iron-dependent programmed cell death characterized by lipid peroxide accumulation, is a newly identified pathway involved in OA pathogenesis. MQC collapse induces ferroptosis by impairing mitochondrial antioxidant capacity (81, 82). Research showed that MQC collapse downregulates SLC7A11 and glutathione peroxidase 4 (GPX4) expression: SLC7A11 mediates cystine transport for glutathione synthesis, and GPX4 scavenges lipid peroxides. Their downregulation leads to lipid peroxide accumulation. Meanwhile, MQC collapse disrupts mitochondrial iron metabolism, increasing cytoplasmic iron levels.

In the OA inflammatory microenvironment, IL-1β/TNF-α upregulate TFR1/DMT1 and downregulate FPN, exacerbating iron overload. Iron ions catalyze ROS production via the Fenton reaction, accelerating ferroptosis (38). Targeting MQC to activate PINK1-Parkin-mediated mitophagy upregulates GPX4/SLC7A11, reducing lipid peroxides and iron accumulation, which inhibits ferroptosis and alleviates cartilage damage. However, chondrocyte specificity of pathway activators in human OA remains unvalidated.

## 6 Characteristic alterations and regulatory divergence in MQC during early-stage OA

The core pathological hallmark of early-stage OA is the disruption of cartilage homeostasis, which involves not only the degeneration of articular cartilage but also a deeper imbalance in cellular metabolism (83, 84). Mitochondria, serving as central hubs for cellular energy metabolism and signal transduction, play a critical role in maintaining chondrocyte function through MQC mechanisms. Studies indicate that characteristic dysregulation of mitochondrial quality control is already evident in early-stage OA, and its regulatory patterns differ significantly from those in middle to advanced stages. This provides novel and crucial targets for understanding the early pathogenesis of the disease and exploring targeted intervention strategies.

### 6.1 Characteristic changes of MQC in early OA

In early-stage OA, MQC undergoes a series of characteristic and compensatory alterations. Dynamically, Drp1-mediated mitochondrial fission is preferentially activated, leading to mitochondrial network fragmentation, while the expression of fusion proteins (e.g., Mfn1/2, OPA1) remains relatively unchanged in the initial phase, reflecting a ‘fission-dominant’ imbalance. Concurrently, mitophagy is compensatorily upregulated via the PINK1/Parkin pathway in early OA models, enabling selective clearance of damaged mitochondria without excessive or exhaustive activation.

Biogenetically, the expression of the master regulator PGC-1α and its downstream pathways retains functional potential, sustaining the capacity for mitochondrial biogenesis (38). Furthermore, mitochondrial proteases (e.g., Lon, ClpXP) exhibit early functional decline, impairing the clearance of misfolded proteins, albeit without triggering widespread protein aggregation at this stage. Mild dysfunction in the electron transport chain results in increased ROS production; however, these species primarily act as signaling molecules that initiate adaptive responses rather than causing irreversible oxidative damage or explosive accumulation (22, 85).

These early, modulatory dysregulations contrast sharply with the advanced-stage manifestations—such as suppressed fusion, mitophagic failure, loss of biogenetic capacity, and uncontrolled oxidative stress—thereby highlighting a critical window and actionable targets for early intervention.

### 6.2 Regulatory differences of MQC and their potential as early intervention targets

MQC in early-stage OA exhibits a distinctive ‘compensatory disequilibrium’ state, characterized by partial retention yet progressive decline of the AMPK/SIRT1/SIRT3/PGC-1α regulatory axis. During this phase, the positive feedback loop between AMPK and SIRT3 remains partially functional, sustaining basal homeostasis through redox balance regulation and autophagy activation (10, 78). Concurrently, mitochondrial calcium load is moderately elevated, but the mPTP remains largely closed, indicating preserved compensatory mechanisms in calcium homeostasis. Unlike the comprehensive collapse of MQC networks in advanced OA, low-grade inflammatory signals (e.g., IL-1β/TNF-α) in early OA may transiently activate certain MQC pathways as an adaptive response.

However, weakened AMPK activation triggers a cascade of deleterious events: reduced SIRT1 activity, diminished PGC-1α-driven mitochondrial biogenesis, and impaired SIRT3-mediated antioxidant defenses. Critically, an early vicious cycle emerges between MQC dysfunction and localized inflammation—inflammatory cytokines suppress core MQC components, while MQC failure-generated ROS and mtDNA further exacerbate inflammation (10, 86).

This dynamic imbalance, oscillating between compensation and decompensation, alongside interindividual variability influenced by genetic and epigenetic factors, defines a critical therapeutic window. Targeting this window—e.g., via specific activation of the AMPK-SIRT3 axis, inhibition of Drp1 hyperactivation-induced fission, or enhancement of PINK1/Parkin-mediated mitophagy—holds promise for restoring mitochondrial metabolic homeostasis and halting OA progression at its initial stages (87, 88). The feasibility of this strategy is preliminarily supported by preclinical studies on bioactive compounds such as curcumin.

## 7 Therapeutic perspectives

In summary, accumulating evidence suggests that MQC dysfunction acts as a potential upstream pathological factor in OA progression, which may be involved in coordinating chondrocyte death, hypertrophy, and senescence via synergistic regulatory networks. Stratified analysis of clinical samples reveals that key MQC molecules (e.g., UPR^mt^ components, PINK1/Parkin) exhibit stage-dependent and site-specific expression patterns in OA cartilage. Specifically, their expression is significantly downregulated in early-stage OA (Kellgren-Lawrence grade I-II) and further decreases with disease progression (grade III-IV); moreover, this downregulation is more prominent in weight-bearing joints (knee and hip) compared to non-weight-bearing joints (finger) (89, 90).

Emerging evidence from clinical cohort studies suggests that MQC-related molecular signatures (e.g., UPR^mt^ activity, mitophagy flux) hold potential as candidate biomarkers for the early diagnosis of OA. These signatures can help distinguish early OA patients from healthy individuals, though large-scale clinical cohort studies and quantitative analyses are still required to verify their sensitivity and specificity. Given the lack of effective OA therapies, targeting MQC has emerged as a novel strategy to halt disease progression (91, 92).

**Figure 2:**
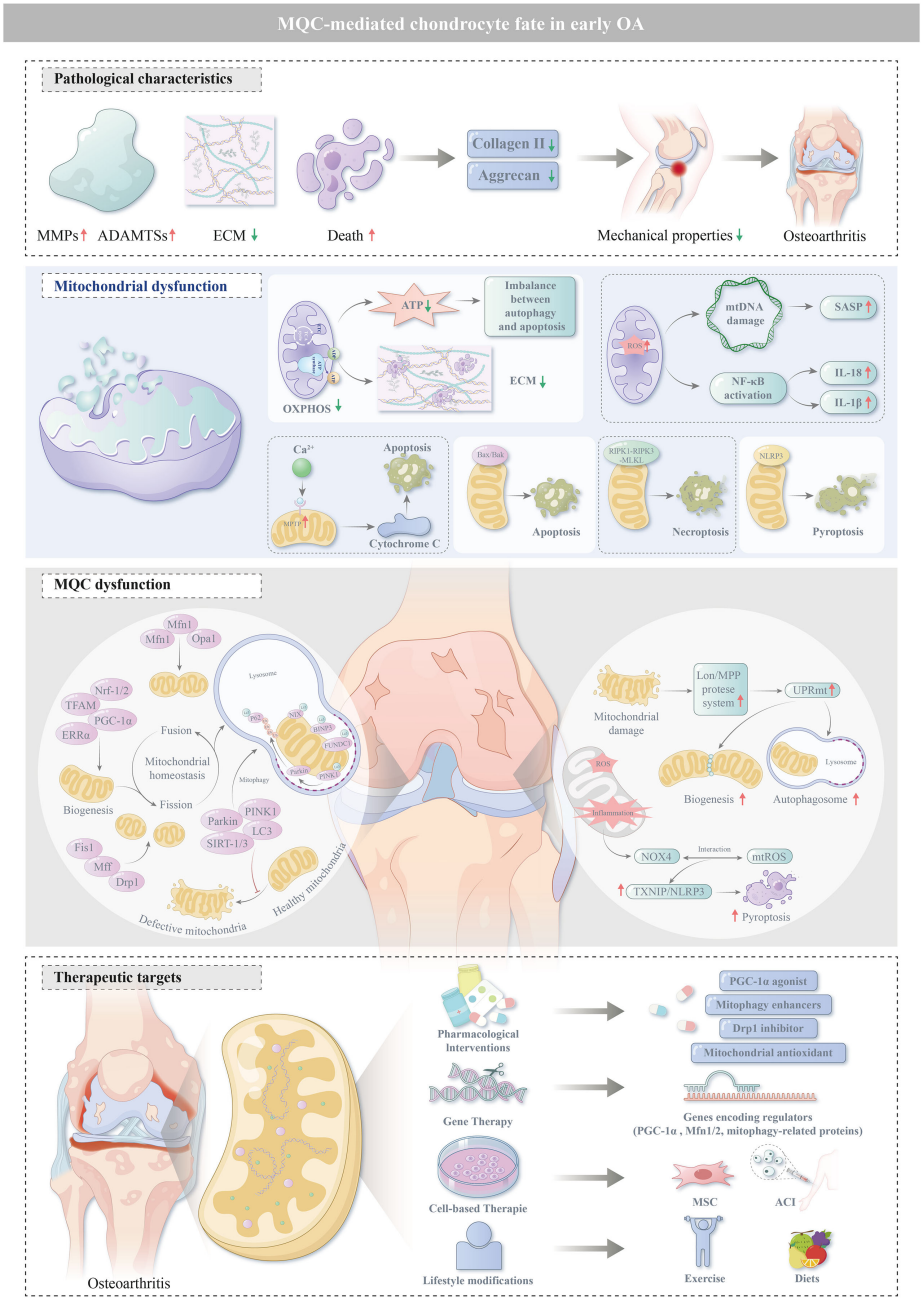
MQC-mediated chondrocyte fate in early OA: This figure systematically summarizes the core pathophysiological characteristics of chondrocytes during the occurrence and development of osteoarthritis, elaborates on the specific manifestations of mitochondrial dysfunction and the molecular mechanism of MQC disorder, and finally focuses on potential therapeutic targets centered on mitochondrial function regulation and MQC disorder correction, providing a clear mechanistic reference and intervention direction for the precise prevention and treatment of osteoarthritis.

Collectively, this review highlights the critical role of MQC in the pathogenesis of OA, uncovers preliminary clinical evidence supporting MQC-related molecular signatures as promising candidate biomarkers for the early diagnosis of OA, and further identifies MQC as a novel, actionable therapeutic target with substantial potential for halting OA progression and improving clinical outcomes. Based on the theoretical framework and preliminary clinical clues summarized above, the subsequent sections will systematically elaborate on the latest research advances in MQC-targeted therapeutic strategies for OA (**Figure 2**).

### 7.1 Pharmacological interventions

Specifically, compounds enhancing mitochondrial biogenesis are of particular interest, with resveratrol and metformin showing notable therapeutic potential. Resveratrol, a natural polyphenol, activates PGC-1α to augment mitochondrial mass and function in chondrocytes (12, 93). *In vitro* studies demonstrated its IC_50_ value is 52.3 μM for OA chondrocytes; *in vivo*, intra-articular injection (50 μM) achieves higher local bioavailability than oral administration (200 mg/kg/d), more effectively restoring MQC and reducing pro-inflammatory cytokines (e.g., IL-1β) in OA models (11). Metformin, an anti-diabetes drug, activates the AMPK pathway to upregulate PGC-1α, with 100-500 μM *in vitro* improving chondrocyte viability; low-dose oral administration (500 mg/kg/d) exerts protective effects via MQC regulation, while high doses exacerbate mitochondrial stress (94). Synergistic effects are observed in combination therapy: Mdivi-1 (Drp1 inhibitor, 10 μM) combined with resveratrol (25 μM) coordinately regulates mitochondrial dynamics (inhibiting fission, promoting fusion) and biogenesis, reducing MMP13 expression by 68% *in vitro* and alleviating cartilage erosion in OA rats more significantly than monotherapy (11, 94).

Metformin, an anti-diabetes drug, activates the AMP-activated protein kinase (AMPK) pathway, upregulating PGC-1α and showing potential in improving chondrocyte viability in OA models. Additionally, mitophagy enhancers such as spermidine derivatives can selectively eliminate damaged mitochondria, complementing MQC regulation to alleviate oxidative stress and apoptosis in chondrocytes. These drug-based interventions hold great potential for halting OA progression by regulating chondrocyte behavior and fate.

### 7.2 Gene therapy

Gene therapy holds promise in targeting MQC genes for treating OA. Viral vectors, such as adeno-associated virus (AAV), are effective in delivering genes encoding crucial regulators like PGC-1α, Mfn1, Mfn2, or mitophagy-related proteins to OA chondrocytes (95). In pre-clinical OA models, AAV-mediated PGC-1α delivery has demonstrated enhanced mitochondrial function and reduced cartilage degradation.

However, significant challenges persist. These include achieving efficient gene delivery to ensure the genes reach a sufficient number of target chondrocytes, precisely controlling long-term gene expression to maintain a stable therapeutic effect, and addressing potential immune responses triggered by the vectors. Overcoming these hurdles could unlock the potential of gene therapy in modulating chondrocyte behavior and fate to combat OA.

### 7.3 Cell-based therapies

Cell-based therapies offer novel approaches for OA treatment. MSCs can modulate the OA joint microenvironment by transferring healthy mitochondria to damaged chondrocytes, which helps restore mitochondrial function in chondrocytes (96). Additionally, MSCs secrete factors that upregulate MQC pathways, promoting cartilage repair in pre-clinical models. This ability to influence the microenvironment and enhance chondrocyte function could potentially halt OA progression. Another strategy is enhancing autologous chondrocyte implantation (ACI). Genetically modifying chondrocytes ex vivo to improve MQC before transplantation can increase their viability and repair capacity in the OA joint (97). However, challenges such as optimizing cell delivery methods and ensuring long-term cell survival need to be overcome to fully realize the potential of these cell-based therapies for OA.

### 7.4 Lifestyle modifications

Lifestyle interventions offer non-invasive strategies that may modulate MQC in OA. For exercise intervention, preclinical and observational studies suggest that low-impact activities (e.g., brisk walking, swimming) with moderate intensity performed for 30 min/d, 5 d/week, may enhance chondrocyte mitochondrial biogenesis via the AMPK/PGC-1α pathway (98, 99). Putative molecular events include AMPK phosphorylation at Thr172, which may directly activate PGC-1α by phosphorylating Ser571, thereby upregulating mitochondrial transcription factors (NRF1, TFAM); in OA chondrocyte models, this cascade has been associated with a 42% increase in mtDNA copy number and a 35% increase in ATP production (99).

Regarding dietary intervention, accumulating evidence indicates that antioxidant-rich foods may exert protective effects via active components at defined concentrations: anthocyanins in blueberries (50-100 μM) and selenium in nuts (0.1-0.5 μM) have been shown to scavenge ROS in *in vitro* models, reducing OA chondrocyte ROS levels by 38-52% and increasing GPX4 activity by 29-41% (100). Additionally, balanced calorie intake (25–30 kcal/kg/d) controls body weight, reducing mechanical stress on weight-bearing joints (101). These quantified lifestyle modifications represent promising, accessible approaches that may support MQC and slow OA progression. However, it is critical to acknowledge that individual variability in treatment response—driven by factors such as age, baseline disease severity, and genetic background—and the paucity of long-term human clinical data specifically evaluating MQC modulation represent key limitations of this approach.

### 7.5 Other therapeutic approaches

Several strategies show promise in OA treatment. Caspase inhibitors like zVAD-fmk have reduced chondrocyte apoptosis and cartilage degradation in rabbit OA models, though their application may be limited to post-traumatic OA and requires precise delivery site control (102). Minimizing oxidative stress (OS) and preserving mitochondrial integrity represent promising alternative therapeutic strategies. Antioxidants exert anti-apoptotic and disease-modifying effects in OA, and autophagy induction can eliminate dysfunctional mitochondria to restore chondrocyte homeostasis. Yet, autophagy modulation requires stringent regulation, as excessive or dysregulated induction may paradoxically trigger chondrocyte apoptosis and exacerbate OA progression (103).

Targeting specific microRNAs (miRs) presents novel therapeutic potential. For instance, miR-142-3p acts as an anti-apoptotic molecule, and its overexpression slows OA progression in mice, suggesting it as a potential target. Conversely, miR-155, an autophagy inhibitor overexpressed in OA, could be targeted with anti-sense strategies to restore autophagy in chondrocytes (104). Preserving subchondral bone integrity also represents a promising therapeutic avenue, given its early and pivotal involvement in OA pathogenesis. Additionally, targeted therapies that attenuate the catabolic phenotype of chondrocytes may confer protective effects against post-traumatic OA (PTOA). A more comprehensive mechanistic understanding of chondrocyte apoptotic and autophagic processes will further facilitate the development of novel, disease-modifying OA treatments.

## 8 Conclusions

OA, a highly prevalent degenerative joint disorder, imposes substantial socioeconomic burdens and causes severe disability worldwide. Its clinical management remains challenging due to the incomplete understanding of its complex multifactorial pathogenesis, which involves the intricate interplay of genetic, environmental, metabolic, and mechanical factors. Accumulating evidence confirms that abnormal chondrocyte behaviors, including disrupted synthetic/catabolic balance and dysregulated cell death modalities, are central to OA progression, while MQC serves as a pivotal regulatory node in maintaining chondrocyte homeostasis and function.

Emerging preclinical and clinical studies have highlighted that MQC dysfunction contributes to mitochondrial damage, excessive ROS production, and accelerated cartilage degeneration, implicating MQC as a potential early therapeutic target for OA. Various MQC-targeted therapeutic strategies, such as enhancing mitochondrial biogenesis, promoting mitophagy, and regulating mitochondrial dynamics, together with combined approaches (e.g., gene therapy, cell-based therapies, and lifestyle modifications), have demonstrated promising protective effects in preclinical OA models. However, several critical limitations currently hinder the translation of these findings and the novelty of MQC-related OA research, which must be addressed to advance the field.

First, direct causal links between MQC defects and human OA pathogenesis remain incompletely validated, as most current evidence is derived from preclinical models rather than well-designed longitudinal human cohort studies. Second, MQC dysfunction exhibits distinct subtype-specific patterns across different OA stages and patient populations, but stratified analyses to define these heterogeneous characteristics are lacking. Third, the clinical translation of MQC-targeted therapies is limited by unclear specific molecular targets, suboptimal treatment regimens, and the paucity of long-term safety and efficacy data in human trials. Fourth, the MQC-centric pathogenic model often overlooks crosstalk with other key OA-related pathways (e.g., matrix metalloproteinase-mediated matrix degradation, inflammatory dysregulation, and mechanical stress), which hinders a holistic understanding of OA heterogeneity and pathogenesis.

To address these limitations and enhance the novelty and translational value of future research, we propose targeted solutions: First, establish large-scale longitudinal clinical cohorts integrating MQC molecular profiling with long-term clinical outcomes to validate causal relationships and identify MQC-related early diagnostic biomarkers; Second, conduct stratified analyses to characterize subtype-specific MQC dysfunction patterns, enabling the development of personalized MQC-targeted therapeutic strategies based on disease stage and individual patient status; Third, optimize translational research pipelines by performing phase I/II clinical trials to evaluate the safety and efficacy of MQC modulators, and explore combinatorial therapies to enhance synergistic effects; Fourth, employ multi-omics and spatial transcriptomics approaches to systematically map the crosstalk between MQC and other critical pathogenic axes, facilitating the development of network-based therapeutic strategies.

In conclusion, integrating MQC-related mechanisms with other key OA pathogenic pathways is essential for a comprehensive understanding of OA pathogenesis. Addressing the current limitations and implementing the proposed solutions will facilitate the translation of preclinical MQC research into clinically actionable interventions, which may ultimately improve OA diagnosis, management, and patient quality of life. Despite significant remaining challenges in clinical translation, MQC holds great promise as a pivotal target for early and personalized OA intervention.
